# *In-Silico* Algorithms for the Screening of Possible microRNA Binding Sites and Their Interactions

**DOI:** 10.2174/1389202911314020005

**Published:** 2013-04

**Authors:** Harsh Dweep, Carsten Sticht, Norbert Gretz

**Affiliations:** 1 Medical Research Center, Medical Faculty Mannheim, University of Heidelberg, D-68167 Mannheim, Germany

**Keywords:** microRNAs, miRWalk, Target prediction, Promoter, CDS, UTR, Prediction algorithm, Database.

## Abstract

MicroRNAs (miRNAs) comprise a recently discovered class of small, non-coding RNA molecules of 21-25 nucleotides in length that regulate the gene expression by base-pairing with the transcripts of their targets i.e. protein-coding genes, leading to down-regulation or repression of the target genes. However, target gene activation has also been described. miRNAs are involved in diverse regulatory pathways, including control of developmental timing, apoptosis, cell proliferation, cell differentiation, modulation of immune response to macrophages, and organ development and are associated with many diseases, such as cancer. Computational prediction of miRNA targets is much more challenging in animals than in plants, because animal miRNAs often perform imperfect base-pairing with their target sites, unlike plant miRNAs which almost always bind their targets with near perfect complementarity. In the past years, a large number of target prediction programs and databases on experimentally validated information have been developed for animal miRNAs to fulfil the need of experimental scientists conducting miRNA research. In this review we first succinctly describe the prediction criteria (rules or principles) adapted by prediction algorithms to generate possible miRNA binding site interactions and introduce most relevant algorithms, and databases. We then summarize their applications with the help of some previously published studies. We further provide experimentally validated functional binding sites outside 3’-UTR region of target mRNAs and the resources which offer such predictions. Finally, the issue of experimental validation of miRNA binding sites will be briefly discussed.

## 1 INTRODUCTION

Functional studies indicate that microRNAs (miRNAs) regulate the expression of crucial genes which are involved in the initiation, progression and prognosis of many human pathologies. miRNAs comprise a recently discovered class of small, non-coding RNA molecules of 21-25 nucleotides (nt) in length that regulate the gene expression by base-pairing with the transcripts of their targets i.e. protein-coding genes, leading to down-regulation or repression of the target genes [1]. However, target gene activation has also been described [2]. miRNAs are involved in diverse regulatory pathways, including control of developmental timing, apoptosis, cell proliferation, cell differentiation, modulation of immune response to macrophages and organ development [1, 3] and are associated with many diseases, such as cancer [3-11].

miRNA transcripts initially originate as long primary miRNAs (pri-miRNAs) by RNA polymerase II. The pri-miRNAs are then processed to a stem-loop (hairpin) structure fragment termed precursor miRNAs (pre-miRNAs) in the nucleus by an RNase III enzyme, Drosha, in complex with the double-strand RNA-binding protein DGCR8 [12, 13]. In a next step, the approximately 70nt hairpin structure pre-miRNAs are then exported to the cytoplasm by Exportin-5, where they are transformed into small, single-stranded miRNAs with the help of Dicer [12, 13]. One strand of the mature miRNA enters the RNA-induced silencing complex (RISC) which contains Argonaute2 (Ago2), Dicer, and transactivating response RNA-binding proteins and binds to the 3’-untranslated region (3’-UTR) of the target mRNA. Whereas, the other strand, termed miRNA*, is degraded. If the complementarity is perfect, then RISC usually cleaves the target mRNA (classical RNA interference); however, if the base-pairing is imperfect, then RISC induces translational repression of target genes by targeting their 3’-UTR [1, 13, 14]. Previously it has been shown that 5’ end of miRNA could be determinant in target repression [15]. The 5’ end sequence of miRNA is called ‘‘seed’’ and has a length of 6-8nt which is energetically favourable for the miRNA-target interaction [16]. Mutations in the seed region of a miRNA sequence leads to a lack of interaction [17]. The binding reduces the expression level of target protein by a number of mechanisms including inhibition of translational initiation [18], inhibition of elongation, and induction of deadenylation which decreases mRNA stability and increases the rate of mRNA degradation [13]. In addition to their role in such posttranscriptional repression, miRNAs are now implicated in transcriptional gene silencing by targeting the promoter region [19]. Thus, miRNAs can inhibit gene expression via translational repression, target mRNA degradation, or transcriptional inhibition.

The miRNA gene family is one of the largest in higher eukaryotes: more than 1,900 human mature miRNAs have been documented in the release 18 of miRBase database [20], each of them having the potential to bind to hundreds of transcripts [21]. Since the breakthrough discovery of the very first miRNAs, computational methods have been proven to be relevant tools in understanding the mode of miRNAs action. Most computational methods applied in miRNA research are about miRNA-mRNA interaction predictions. Few years ago, scientists determined miRNA-mRNA interactions through experiments. The first miRNAs and their target genes were identified by genetic techniques [22]. Nonetheless, these genetic techniques were abandoned due to the lack of high-throughput experimental methods and laborious nature; therefore, it is of paramount importance to design computational approaches to identify miRNA-target interaction predictions. In this review, we summarize the rules adapted by prediction algorithms to generate possible miRNA binding site interactions and to introduce most relevant methods that have been developed for miRNA-mRNA interactions prediction. We then outline the applications of these most relevant algorithms with the help of previously published studies. We further provide experimentally validated functional binding sites outside 3’-UTR region of target mRNAs and the resources which offer such predictions. Finally, the issue of experimental validation of miRNA binding sites will be briefly covered.

## 2 PREDICTION CRITERIA FOR miRNA TARGET IDENTIFICATION

miRNA binding sites screening principles adapted by the most algorithms are relatively similar, and are based on previous knowledge on the pairing of mRNAs and miRNAs [22]. Prediction criteria used by animal miRNA targets prediction algorithms include the following:

### 2.1 Base Pairing

The target prediction programs identify potential miRNAs binding sites within the mRNA 3’-UTR sequence according to specific pairing patterns. These sites can be classified into six categories: (i) 5’-dominant canonical [23], (ii) 5’-dominant seed only [23], (iii) 3’-compensatory [23], (ív) newly discovered central pairing sites [24], (v) miBridge (simultaneous 5’- and 3’-UTR sites) [25] and (vi) Pivot [26]. The possible interactions of these six sites are shown in Fig. (**1**). miRNA seed is defined as the consecutive 7 to 8nt long sequence starting from either the 1^st^ or 2^nd^ position at the 5’ end of a miRNA [16, 27]. In addition to the 3’-UTR region, other algorithms, like miRWalk [27], identify multiple consecutive Watson-Crick complementary subsequences between miRNA and the complete sequence (10kb upstream flanking region assumed promoter, CDS, 5’- and 3’-UTR) of a gene.

### 2.2 Thermodynamic Stability of miRNA-mRNA Duplex

The kinetics and thermodynamics (minimum free energy, MFE) of RNA-RNA duplexes can be determined by RNA folding programs, and have been considered important by most algorithms. Nonetheless, a recent study by Lewis *et al.* [28] has demonstrated that thermodynamics can be omitted without lowering the specificity of the detection algorithm by integrating other conserved sequence information.

### 2.3 Conservation of Target Sites

Comparative sequence analysis within related species is performed to examine if target sequences are evolutionarily conserved across species. In order to reduce the number of false positives, many target prediction algorithms scan orthologous 3'-UTR sequences and then perform conservation analysis across related species. However, there are issues associated with conservation analysis. The use of conservation filter has a risk of increasing false negatives whereas it reduces false positives.

### 2.4 Cooperative Translational Control and Multiplicity of miRNA Binding Sites

Multiple miRNAs typically regulate one mRNA. The multiple miRNAs binding site in the same region of a gene can potentially increase the level of translational suppression and enhance the specificity of gene regulation, whereas one miRNA may have several target genes, reflecting target multiplicity. That is, combinatorial control of a single target by multiple miRNAs may be an important feature of miRNA targeting and multiple binding sites for a miRNA within the mRNA 3’-UTR region can increase the efficiency of RNA silencing [17]. Thus, some algorithms scan for the presence of multiple target sites [27, 29].

## 3 ALGORITHMS FOR ANIMAL miRNA-TARGET PREDICTIONS

Computational prediction of miRNA targets is much more challenging in animals than in plants, because animal miRNAs often perform imperfect base-pairing with their target sites, unlike plant miRNAs which almost always bind their targets with near perfect complementarity. In the past years, a large number of target prediction programs have been developed for animal miRNAs. These miRNA-target prediction algorithms are commonly based on a base-pairing rule, and other features such as evolutionary conservation, thermodynamics of mRNA-miRNA duplexes and nucleotide composition of target sequences are often integrated to improve the accuracy. Currently existing miRNA-target predictions algorithms are shown in (Table **1**) and the most relevant programs out of them are briefly described below.

### 3.1 DIANA-microT

This algorithm was developed by Kiriakidou *et al.* [30] by amalgamating computational and experimental approaches. For the screening of putative miRNA-recognition elements (MREs), it uses a 38nt long frame that is progressively moved along 3’-UTR. The minimum energy of potential miRNA-target interaction is calculated at each step by using dynamic programming that allows mismatches and is compared with the findings from scrambled sequences with the same dinucleotide content as real 3’-UTRs. DIANA-microT recognizes 7, 8 or 9nt long complementary seeds from the 5’ end of miRNA sequence with canonical central bulge within the analyzed 3’-UTR. Hexamer sites within the seed region or with one wobble pairing are also considered while these results are enhanced by additional base pairing in 3’ region of miRNA [31]. DIANA-microT adapts conservative alignment for scoring but also considers non-conservative sites. It also provides users with a percentage probability of existence for each result depending on its pairing and conservation profile.

### 3.2 miRWalk

The miRWalk algorithm [27] is a recently designed computational approach which identifies multiple consecutive Watson-Crick complementary base-pairings between miRNA and gene sequences. This algorithm searches for seeds based on Watson-Crick complementarity, walking on the complete sequence of a gene starting with a heptamer (7nt) seed from 1^st^ and 2^nd^ position from the 5’ end of miRNA sequences. As soon as it identifies a heptamer perfect base-pairing, it immediately extends the length of the miRNA seed until a mismatch arises. It then returns all possible hits with 7nt or longer matches. These binding sites are then separated on the basis of their identified locations (start, and end positions and regions) in the analyzed sequences. Then it assigns the prediction results in five parts, according to promoter region, 5’-UTR, CDS, and 3’-UTR and mitochondrial genes. In addition, the probability distribution of random matches of a subsequence in the analyzed sequence is calculated by using Poisson distribution [32]. It can be expected that the longer perfect complementation of a seed is associated with a lower probability, thus the higher are the chances of an effective miRNA-target interaction.

### 3.3 miRanda

The miRanda algorithm [33] was originally created to identify the putative miRNA target genes in Drosophila melanogaster. However, it was also applied to predict miRNA-target interactions in humans. It is based on a comparison of miRNAs complementarity to 3’-UTR regions. Three rules (sequence complementarity based on a position weighted local alignment algorithm, free energies of RNA-RNA duplexes and conservation of target sites in related genomes) are used to select the target genes for each miRNA. In the improved version of miRanda [34], one wobble pairing is allowed in the seed region that is adjusted by matches in the 3’ end of the mature miRNA sequence. This strategy is adapted to integrate different types of miRNA-target interactions. miRNAs with multiple binding sites within 3’-UTR are promoted, which participates in increasing the specificity, however, it diminishes the number of miRNAs with a single but perfect base pairing. It takes into account the evolutionary relationships of interactions more globally focusing on the conservation of miRNAs, relevant parts of mRNA sequences and the presence of a homologous miRNA-binding site on the mRNA [35].

Recently, an important improvement to the miRanda algorithm has been introduced [36]. Betel *et al.* designed a support vector regression (SVR) based novel algorithm, called mirSVR, for scoring and ranking the miRNA-target interactions resulting from the miRanda algorithm by adopting supervised learning on mRNA expression changes following miRNA transfections. mirSVR combines target site information and contextual features into an integrated model. Notably, this method detects a significant number of experimentally observed non-canonical and non-conserved sites [36].

### 3.4 PicTar

PicTar [37, 38] identifies binding sites that are co-regulated by multiple miRNAs in a synergistic manner, in addition to binding sites targeted by a single miRNA and then calculates the free energy of identified miRNA-target duplexes. Each finding is scored using a Hidden-Markov Model (HMM). miRNAs with multiple alignments are preferred. PicTar uses genome-wide sequence alignment of eight vertebrate species to eliminate false positive results and it scores the candidate genes of each species separately to calculate a combined score for a gene. It is mandatory to have recurring nucleotides at overlapping positions in the analyzed mRNAs of paired species.

### 3.5 PITA

The PITA [39] algorithm predicts miRNA-target interactions by focusing on the accessibility of the target sequence (mRNA) that is specifically affiliated with the secondary structure of the analyzed transcript. PITA works on an assumption which is based on the fact that the secondary structure of the mRNA plays an important role in miRNA-target interaction recognition by thermodynamically promoting or preventing the interaction. It first scans the complementarity seeds (single mismatch or G:U wobble pairing can be allowed) of miRNAs within the sequences of the analyzed mRNAs and then compares the miRNA-target duplex free energy, and the energetic cost of impairing the target to make it accessible to the miRNA.

Previous studies have also incorporated the folded mRNA secondary structure information for the possible screening of miRNA-target interactions [40, 41]. Long *et al.* [40] designed a two step model i.e. nucleation potential and hybridization energy for a miRNA-target interaction. By considering the role of target secondary structure on the efficacy of repression by miRNAs, they employed Sfold, a RNA secondary structure prediction program, for the statistical analysis of the structures that co-exist in dynamic equilibrium for a particular mRNA. Furthermore, Long *et al.* conducted a comparative analysis of the binding sites of miR-122 within the genome of Hepatitis C virus (HCV) to classify the functional (5’ non-coding region site) and nonfunctional (3’ non-coding region site) interactions. Their model well explained the difference between these two sites, which were not, justified by several miRNA-target prediction algorithms i.e. TargetScan, PicTar, miRanda and RNAhybrid. On the other hand, Robins *et al.* [41] developed an algorithm for target prediction by integrating the RNA structure information, the 7nt from 5’ end, the entire miRNA match score and combined scores for multiple sites in the targets, but they did not consider evolutionary conservation. Their findings suggest that miRNAs have fewer targets than previously reported which is contrary to other studies [28, 42, 43].

### 3.6 RNA22

Rna22 [44] detects pre-created miRNA patterns (statistical significant miRNA motifs that are generated by analyzing the known mature miRNAs sequence) in the analyzed mRNA sequence. It first searches for reverse complement sites of patterns within mRNA of interest and determines sites with many patterns aligned (so called ‘hot spots’). In a next step, it identifies those miRNAs that are likely to anneal with these sites. This approach also allows identifying sites targeted by yet-undiscovered miRNAs. The important parameters (such as minimum number of base-pairs between miRNA and mRNA, the maximum number of unpaired bases and the free energy cut-off) are defined by users. Rna22 does not account for the cross-species conservation criteria in the final scoring.

### 3.7 RNAhybrid

RNAhybrid [32] is an improved version of classical RNA secondary structure prediction programs such as Mfold [45, 46] and RNAFold [47]. This program identifies the energetically most favourable binding sites of a small RNA within a large target RNA sequence. RNAhybrid detects miRNA targets by matching a 6nt seed starting from the 2^nd^ position from the 5’ end of the mature miRNA sequence.

### 3.8 TargetScan and TargetScanS

Both algorithms [28] rely on different approaches for the prediction of possible miRNA-target interactions. TargetScan [16] identifies a perfect Watson-Crick base pairing complementary between 7nt long miRNA seed (from base 2 to 8 in the 5’ end of the miRNAs), and the annotated 3’-UTR sequence and expends each seed match with additional base pairings to the miRNA. It further calculates the thermodynamic free energy of miRNA-target interaction using the RNAFold package [47]. Then it assigns a score to each UTR. Thereafter, it scans the sets of UTRs from other species (such as mouse and rat) for phylogenic analysis.

TargetScanS [28] is an alternative, simplified version of TargetScan which predicts targets having a conserved 6nt seed match flanked by either a 7nt match or 6nt with A on the 3’ terminus not taking into consideration the free energy values.

By integrating both computational and experimental approaches, Grimson *et al.* constructed a model for predicting effective miRNA sites based solely on five features: local AU content, cooperativity of sites, proximity to nucleotides pairing to miRNA (from position 13 to 16), location of sites from the stop codon and location away from the center of long UTRs [48]. Using univariate regression between feature scores and expression changes, they developed a scoring system named “context score”, which has been integrated to TargetScan. Also, this model was experimentally validated for both exogenous and endogenous miRNA-target interactions as it accurately distinguishes effective from non-effective sites without considering evolutionary conservation filter.

## 4 APPLICATIONS OF MOST RELEVANT RESOURCES

The information stored in these databases has been utilized by the scientific community in different ways. A few examples are:

- Some research groups have been adopting a new approach for the identification of new targets for known miRNAs. In this approach, miRNA expression in healthy and/or diseased tissue and/or organ was profiled by using miRNA microarrays and statistical significant miRNAs were then selected for further validation by Northern blot and/or qPCR (quantitative real-time polymerase chain reaction) experiments. Afterwards, miRNA-target prediction programs were interrogated to identify the possible target genes of these miRNAs [49]. Finally, cell lines and/or animals were used for the knockdown, knockout or overexpression of these miRNAs to measure the expression level of putative targets (genes). miRWalk database is helpful for this kind of approach, as a user can easily retrieve information on possible miRNA binding sites within the complete sequence or specific region(s) of targets by supplying miRNA names or uploading a file under the ‘Predicted Target module’ (as shown in many articles for e.g. [50]).
- The basic information on miRNAs (like mature, and stem loop sequence, identifiers, chromosome, strand and band) as well as other necessary data required for a miRNA research, such as regulatory binding sites on upstream and/or downstream flanking regions of pre-miRNA, information on the host gene of miRNA, which miRNAs share a similar seed with the user input miRNAs can be easily obtained from miRBase, miRWalk and miRDB.
- One can collect the putative mRNA-miRNA interaction pairs within other regions of genes from miRWalk database and then validate new targets of miRNA on the other regions of their genes of interest as utilized in [51].
- Few resources such as miRWalk, TargetScan, miRanda, and TarBase and miRTarBase are very helpful for those researchers who want to collect the predicted as well as the experimentally validated miRNA binding site information on their genes (obtained from mRNA microarrays profiling) of interest. They can easily interrogate these databases to gather such information by uploading a file of gene symbols or EntrezIDs. Thereafter, the potential candidates out of these predictions can be selected to conduct further analysis/experiments as described in [52].
- The comparative platform of miRWalk database has been extensively used and described in many articles (e.g. [53]), since it is very useful for carrying out a comprehensive analysis of miRNA binding sites resulting from 10 different prediction datasets and also helpful in reducing the false positives.
- A recent article [54] demonstrated a novel layer of crosstalk between nucleus and mitochondria through a specific subset of human miRNAs by using the putative miRNA binding sites from miRWalk [27], RNA22 [44], RegRNA [55] and TargetScan [16, 28] databases.
- Three recently published articles on miRNA regulations describe the utilization of miRWalk and TargetScan predictions in copy number variants [56], different splice variants of a gene [57] and single nucleotide polymorphisms (SNP) [53].
- Moreover, the experimentally validated miRNAs (e.g. [58]) on one or more genes (for example [59]), diseases, organs and OMIM disorders provide possible suggestions for the treatment of diseases by miRNA (s). The information on cell lines related to specific miRNA(s), diseases, and gene(s) can be easily retrieved from miRWalk [27], TarBase [60], miRTarBase [61], miR2Disease [62], PhenomiR [63] and HMDD [64]. Afterwards, these cell lines can be used to measure the expressions of one or more predicted miRNAs and/or genes under different pathophysiological conditions. Recently, Angerstein *et al.* [65] illustrated a schematic workflow on how to employ the existing miRNA-target interaction resources in identifying the regulatory roles of miRNA in multiple sclerosis. The authors utilized the documented information for analysing the miRNA-target interaction network map, possible regulatory effects of miRNAs on cellular functions and their regulation by transcription factors resulting in tissue-specific expression. Their comprehensive workflow pipeline serves as a useful guideline for performing similar studies on different human pathologies.

## 5 miRNA BINDING SITES OUTSIDE 3’-UTR REGION

In animals, it is well accepted that miRNAs control their target gene expression through base-pairing within 3’-UTRs regions of mRNAs. Therefore, for more than a decade, attempts to identify the miRNA-target interactions have been focused to 3’-UTR regions. However, recent experimental studies on miRNA-target interaction have revealed a novel miRNA mechanism through which they may regulate the gene expression by targeting promoter as well as CDS regions. Tay *et al.* described the natural occurring binding seeds of miR-314, miR-296 and miR-470 within the CDS region of the genes Nanog, Oct4 and Sox2 [66]. On the other hand, a few experiments have indicated possible target sites within the 5’-UTR [67]. A few other studies have revealed that small RNAs positively regulate target sequences. Kuwabara *et al.* reported a small RNA isolated from neural stem cells that can transcriptionally activate the expression of genes harbouring NRSE/RE1 sequences [68], Jopling *et al.* discovered a liver specific miRNA which enhances viral replication by annealing to the 5’ noncoding region of the viral genome [69], Li *et al.,* have demonstrated several dsRNAs that activate the expression of three genes (E-cadherin, p21 and VEGF) by targeting their noncoding regulatory regions in gene promoters [2] and Place *et al.* have shown that miR-373 targets the promoter sequences of E-cadherin and CSDC2 genes to induce gene expression [70]. These findings suggest that one or more miRNAs could positively regulate the expression of many genes by targeting their inhibitors (repressors of the upstream flanking region or promoter sequence). These kinds of interactions (miRNA-mediated activation of genes) have recently been confirmed in a number of studies [49, 71, 72].

Kim *et al.* accomplished an in-silico screening for miRNA binding sites proximal to known gene transcription start sites in the human genome [19]. An evidence of a *cis*-regulatory role of miR-320 (located within the promoter region of POLR3D) in transcriptional silencing of POLR3D (a cell cycle gene) expression was also confirmed [19]. The authors proposed a potential role of endogenous miRNA pathway in transcriptional and epigenetic gene silencing [19]. For such miRNA-directed gene silencing, it is possible that miRNAs enter the nucleus to undergo modifications or to associate with nuclear localized proteins. Guang *et al.* have shown that Argonaute proteins can transport classes of small regulatory RNA to distinct cellular compartments to regulate gene expression [73]. It is also possible that miRNAs are involved in chromatin remodeling [19] or associate with target transcripts in the nucleus. Within the last few years, there have been few studies revealing nuclear import of miRNA [74] or their localization within ribosome-rich regions in both the nucleolus and cytoplasm [75]. Taken together, these findings suggest that animal miRNAs could modulate the expression of their targets (silencing or activating) by annealing to any region on the gene sequences. Based on such evidence, we have recently developed miRWalk [27] which is so far the only database which hosts the putative miRNA binding sites not only within 3’-UTR regions, but also in the other regions (promoter, 5’-UTR and CDS) of a gene. We assume that these binding regions could also be considered as a potential target of miRNAs and such findings could facilitate researchers to validated new targets of miRNA not only within the mRNA 3’-UTR, but also in the other regions of a gene.

## 6 FUNCTIONAL EXTENSION OF miRNA-TARGET PREDICTION RESOURCES

Many web-based resources have been hosted integrating novel features to existing prediction programs. These databases mostly incorporate the putative miRNA-target information resulting from already established prediction programs and include other necessary data that are associated with many miRNA, gene, protein or pathway resources such as UCSC genome browser, NCBI, miRBase, Ensembl, Swiss-Prot, KEGG pathway and other databases.

Similar to miRGator [76], which integrates three prediction databases, miRWalk integrates 10 datasets for a comprehensive study. The miRWalk database is the most frequently utilized resource to conduct a comparative analysis of miRNA binding sites resulting from the miRWalk algorithm and 8 other prediction programs. This resource also hosts the experimentally validated information which is collected by an automated-text mining search as well as from existing databases that document miRNAs validated data.

TarBase [60] and miRTarBase [61] document the experimentally verified information on miRNA-target interactions along with their validation methods such as reporter genes, qPCR, western blotting, microarrays, proteomics, sequencing and degradome sequencing data. Such information is manually curated by both resources through reading each and every page of the current literature in PubMed and hosted as experimentally validated miRNA-target interaction data. miR2disease [62], PhenomiR [63] and miRWalk [27] resources provide miRNA-disease and miRNA-cell line interactions. The miRDB is also a web-based resource for target prediction and functional annotation [77]. An editable Wikipedia interface is integrated to miRDB for hosting the functional annotations of miRNA which can be easily corrected, edited or updated by miRNA community. In addition to wiki annotations, it also has a section which hosts target predictions and basic information of miRNAs.

## 7 WHICH DATABASE IS BETTER FOR miRNA-TARGET PREDICTIONS?

Comparative studies conducted with the earlier miRNA-target prediction programs suggested that no program was consistently superior to all others [78, 79]. Indeed, it has become a common practice for the experimental researchers to look at predictions produced by several miRNA-target interaction algorithms and focus on their intersection [49, 53, 78]. The resources (for instance, miRWalk) that host predictions produced by using multiple algorithms might be helpful to reduce the probability of introducing false positives and/or negatives as much as possible. miRWalk [27] integrates 10 datasets for a comprehensive study of the putative miRNA binding site predictions obtained from different algorithms. This resource allows the user to take more control over the prediction data, they consider. This resource conveniently incorporates eight different databases at one place. It also allows users to choose which combinations of databases they would like to consider for their search. In addition, miRWalk database also supplies a more holistic view of genetic networks of miRNA-gene-pathways and miRNA-gene-OMIM disorder interactions, and hosts new and unique features on experimentally validated miRNAs. Besides validated information, it also offers the information on proteins known to be involved in miRNA processing and provides available literature on miRNAs. Other programs such as TargetScan [16, 28], miRanda [33-35] and PicTar [37, 38] are also employed to search conserved miRNA binding sites. Table **2** displays the special features of popular miRNA-target prediction algorithms.

## 8 EXPERIMENTAL VERIFICATION OF PUTATIVE TARGET SITES

Once the potential putative miRNA-target interaction pairs are obtained, the next step is to experimentally verify these predicted miRNA binding sites. Since miRNA-target prediction algorithms are not perfect, there is always a fair possibility of false-positive predictions associated with them. Therefore, the experimental validation of these predicted miRNA-target interactions in the biological system is necessary to complete the study of target prediction. Several methods such as reporter assays, microarray and proteome analyzes for experimental verification of predicted miRNA-mRNA interactions are currently being utilized. The experimentally validated miRNA-target interactions information have been documented in various databases, such as TarBase [60], MiRecords [80], miRWalk [27], miRTarBase [61] and miRNAMAP [81].

A reporter gene assay is the very first method in the field of experimental verification of putative miRNA-target interactions directly, because miRNA activity on such reporter genes can be easily measured [37, 44]. The putative miRNA binding sites are fused to a reporter construct. The mutated miRNA binding sites are also inserted into the constructs which can further use as a negative control to correctly measure the reporter activity. The reporter expression is then measured in the presence and absence of the cognate miRNAs in transfected cell system. Cell systems not expressing miRNA of interest may be co-transfected with the reporter construct and either a miRNA mimetic or a miRNA-encoding vector. This method still serves as an invaluable approach for the validation of individual miRNA-target interactions [53]. The main advantage of this method is its simplicity, whereas the fact that it does not support a high-throughput identification of miRNA-target interactions is an important drawback.

Microarray and pSILAC (stable isotopic labelling with amino acids in cell culture) are highly efficient methods to measure the global transcriptome [82] or proteome [83] changes due to overexpression or silencing of miRNA. However, these methods only provide indirect evidence about miRNA-target interactions and fail to distinguish direct from the indirect targets. Degradome analysis [84, 85] approach is also adapted, but it only works in a system where a miRNA induces RISC-mediated mRNA cleavage, and thus, its usage is restricted mostly to plants.

Other experimental verification of miRNA-target interaction approaches have been suggested and successfully implemented (reviewed in [86]). AGO proteins of RISC can bind both miRNAs and mRNAs and this feature was employed in co-immunoprecipitation assays [87]. Moreover, high-throughput sequencing of RNAs isolated by crosslinking immunoprecipitation (HITS-CLIP) was also applied to determine AGO-bound miRNA-mRNA interaction maps [88]. Such an approach is useful in reducing the false positive targets. The transcriptome-wide identification method i.e. PAR-CLIP (Photo-activated-Ribonucleoside-Enhanced Crosslinking and Immunoprecipitation) which is an improved version of CLIP was used to detect miRNPs (the RNA-binding protein and miRNA target sites) complexes [89]. CLIP are very modern and elegant methods to conduct large-scale analyses, however, they have some weaknesses. These methods are technically challenging and expensive as well as they are unable to distinguishing between direct and indirect miRNA-target interactions. Moreover, Davis *et al.* proposed RNA-ligase-mediated (RLM) 5’ RACE experiments to verify miRNA-target interactions [90], and Li *et al.* determined the genes that are likely to be modulated by miRNAs using high cytoplasmic-to-nucleic ratio of mRNA expression [91]. These two methods were successfully established for the experimental validation of miRNA-target interactions.

## 9 CONCLUDING REMARKS

After the breakthrough discovery of the very first miRNAs and their targets, many miRNA-target prediction algorithms have been developed based on different principles such as base-pairing pattern, evolutionary conservation, secondary structure and nucleotide composition. Since then, there has been a linear growth in the number of annotated miRNAs and their validated or predicted targets which are supported by a large number of miRNA-target prediction programs. Although these programs are still lacking sensitivity and specificity, no program is proven superior to others. Additionally, the methods (for e.g., miRWalk) that provide a comprehensive atlas of the putative miRNA binding site predictions resulting from multiple algorithms are attracting more attention of the scientists who want to consider all the possible combinations (union or interaction) of available algorithms for their research. Since existing target prediction algorithms rely on different assumptions, combining the results from multiple tools seems to be a good practice and is often utilized in reducing the false positives and/or negatives as much as possible. As the understanding of miRNA regulatory mechanism widens, it can be expected that existing algorithms will become progressively more accurate.

## Figures and Tables

**Fig. (1):**
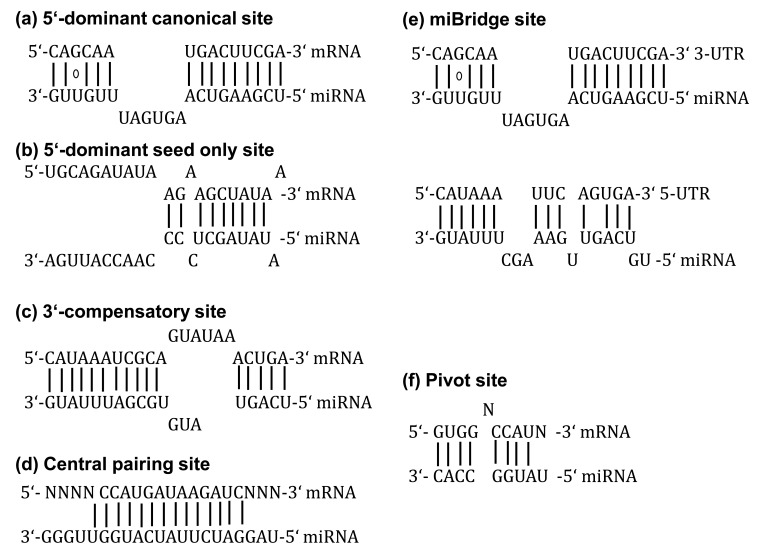
Overview of the possible interactions of six different types of miRNA binding sites.

**Table 1.** Overview of the Existing Resources for Validated and Predicted miRNA-target Information

Content	Resource	URL [Reference]
General information	miRBase	http://www.mirbase.org [20, 61]
Validated miRNA-target interaction information	MiRecords	http://mirecords.biolead.org [80]
	miRTarBase	http://mirtarbase.mbc.nctu.edu.tw [61]
	TarBase	http://www.microrna.gr/tarbase [60]
Predicted miRNA-target interaction information	Diana-microT	http://diana.cslab.ece.ntua.gr/microT [30, 31]
	miRDB	http://mirdb.org [77]
	miRWalk	http://mirwalk.uni-hd.de [27]
	miRanda	http://www.microrna.org [33-35]
	miTarget	http://cbit.snu.ac.kr/~miTarget [92]
	PicTar	http://pictar.mdc-berlin.de [37, 38]
	PITA	http://genie.weizmann.ac.il/pubs/mir07 [39]
	RNA22	http://cbcsrv.watson.ibm.com/rna22.html [44]
	RNAhybrid	http://bibiserv.techfak.uni-bielefeld.de/rnahybrid [32]
	TargetScan	http://www.targetscan.org [16, 28]
miRNA-disease interaction information	HMDD	http://cmbi.bjmu.edu.cn/hmdd [64]
	miR2Disease	http://www.mir2disease.org [62]
	PhenomiR	http://mips.helmholtz-muenchen.de/phenomir [63]

**Table 2.** Overview of the Features of Popular miRNA-target Prediction Programs

Programs	Species	Algorithms	Advantages	Disadvantages
Diana-microT	Human	Seed match, thermodynamics	Prefers target structure before seed pairing.	Absence of cooperativity and multiplicity of miRNA binding sites. Conservation filter.
miRDB	Human, mouse, rat, dog, chicken	SVM classifier	Editable Wikipedia based interface for functional annotation.	Feature selection procedure is missing.
miRWalk	Human, mouse, rat	Seed match, statistical model	Provides binding sites within promoter, 5’-UTR, CDS, and 3’-UTR regions. Amalgamates 10 prediction datasets and other unique features such as validated information on miRNAs associated with genes, diseases, cell lines, pathways, etc.	Free energy of the duplex is missing, however; it integrates other algorithms which consider free energy.
miRanda	Human, mouse, rat, fly, worm	Complementarity, free energy, conservation	Offers tissue-based miRNA expression profile.	Low precision. Conservation filter.
miTarget	Any	Seed match, free energy, SVM classifier	Validated miRNA targets information collected from literature search is used as training dataset.	A simple filtering for feature selection method.
PicTar	Vertebrates, flies, worms	Seed match, free energy, conservation, HMM	Considers cross-species conservation to reduce false positives.	Non-conservative sites prediction.
PITA	Human, mouse, fly, worm	Seed match, free energy	Considers secondary structure for prediction.	Low efficiency to existing algorithms.
RNA22	Human, mouse, fly, worm	Pattern recognition	Serves interactive exploration. It does not consider cross-species conservation filter.	Low efficiency to existing algorithms.
RNAhybrid	Any	Seed match, free energy, statistical model	Extension of classical RNA secondary structure programs.	Unable to distinguish functional and non-functional sites.
TargetScan	Mammals, flies, worms, fish	Seed match, free energy, conservation	Broadly scans for conserved 8nt and 7nt sites.	Restricts to seed matching and conservation.
